# Unusual non-fluorescent broad spectrum siderophore activity (SID EGYII) by *Pseudomonas aeruginosa* strain EGYII DSM 101801 and a new insight towards simple siderophore bioassay

**DOI:** 10.1186/s13568-016-0192-1

**Published:** 2016-03-25

**Authors:** Amira M. Embaby, Yasmin Heshmat, Ahmed Hussein

**Affiliations:** Department of Biotechnology, Institute of Graduate Studies and Research, University of Alexandria, 163 Horreya Avenue, Chatby, P.O. Box 832, Alexandria, 21526 Egypt; Department of Chemistry and Biochemistry, Texas Tech University, TX Lubbock, USA

**Keywords:** *Pseudomonas aeruginosa* strain EGYII DSM 101801, Non-fluorescent SID EGYII, Simple inexpensive bioassay, Siderophore arbitrary units (SAU), Broad spectrum antimicrobial activity and pSID/EGYII

## Abstract

Present study highlights an unusual non-fluorescent hydroxamate broad spectrum siderophore (SID EGYII) activity from *Pseudomonas aeruginosa* strain EGYII DSM 101801, a soil bacterial isolate, along with simple low cost effective siderophore bioassay. Detection of SID EGYII activity qualitatively was proved by masking this activity against *Erwinia amylovora* strain EGY1 DSM 101800, an indicator strain, in well-cut diffusion assay containing 100 µM FeCl_3_. SID EGYII activity was expressed quantitatively as arbitrary units [Siderophore arbitrary units (SAU)] 380 SAU/mL against *E. amylovora* strain EGY1 DSM 101800. Maximal SID EGYII activity was achieved upon growing *P. aeruginosa* strain EGYII DSM 101801 in PYB broth at 180 rpm for 24 h. SID EGYII displayed a broad spectrum antimicrobial activity against some human pathogens (i.e., Gram-positive bacteria, Gram-negative bacteria and yeasts) and a fireblight plant pathogen. Interestingly, transformants of *Escherichia coli* JM109 (DE3)pSID/EGYII harboring *P. aeruginosa* strain EGYII DSM 101801 plasmid demonstrated a perceivable antimicrobial activity against *E. amylovora* strain EGY1 DSM 101800. The broad spectrum antimicrobial activity of the unusual non-fluorescent SID EGYII would underpin its high potential in targeting bacterial pathogens posing probable threats to human health and agricultural economy. The present simple low cost effective bioassay is a new insight towards an alternative to the expensive cumbersome siderophore Chrome Azurol S assay.

## Introduction

Biosynthesis and extracellular secretion of siderophores are one of the sophisticated strategies developed by bacteria to fulfill their iron requirements under circumstances where, the concentration of the growth essential element iron in the environment is below the threshold level required for bacterial growth (Neilands 1981; Crosa 1989). Siderophores are low molecular weight iron scavenging molecules (Neilands 1995; Reimmann 2012) interchangeably called iron uptake mediated systems and certain membrane receptors are responsible for internalization of these iron sequestering complexes inside the bacterial cells (Wandersman and Delepelaire 2004). However, siderophores are not the only route for mediating iron transport inside bacterial cells. Pathogenic bacteria lacking the capability to produce siderophores have protein receptors that could recognize the iron protein complexes to mediate iron internalization inside the bacterial cells (Mickelsen and Sparling 1981; Sebulsky et al. 2000). Several reports addressed siderophores production from different bacteria and fungi as virulence factors (Payne 1983; Tolmasky et al. 1988; Milagres et al. 1999; Ma et al. 2000; Wandersman and Delepelaire 2004; Xianmei et al. 2011; Balado et al. 2015; Prathyusha et al. 2015; León-Sicairos et al. 2015). Reportedly, fluorescent siderophores are produced by some species of fluorescent Pseudomonads whilst, non-fluorescent siderophores are traced in non-fluorescent Pseudomonads (Albesa et al. 1985; Meyer et al. 2002; Visca et al. 2007; Unni et al. 2014). Among well studied siderophores are those produced by fluorescent Pseudomonads particularly *Pseudomonas aeruginosa*. So far, a few number of reports highlighted non-fluorescent siderophore production from fluorescent Pseudomonads (Bultreys et al. 2001; Unni et al. 2014; Mehri et al. 2011). At most, genes encoding siderophore production in bacteria are harbored by plasmids (Tolmasky et al. 1988; Osorio et al. 2015).

As a matter of fact, siderophores display numerous potential applications in medical, agricultural, industrial and pharmaceutical sectors (Vértesy et al. 1995; Hershko et al. 2002; Chua et al. 2003; Husen 2003; Ali and Vidhale 2013; Miethke and Marahiel 2007). In siderophores-antibiotics conjugate studies, the antimicrobial activity imposed by the siderophore component encountered in this complex is always not considered (Nagoba and Vedpathak 2011). From another side, the universal siderophore Chrome Azurol S (CAS) assay is expensive, tedious, time consuming and may be toxic to some microorganisms.

Much attention has been paid to seek for novel siderophores with promising characteristics for better medical and industrial exploitation. The present research aims to search for a new siderophore with wide spectrum antimicrobial activity. The goal extends to tailor a new simple low cost effective method for both qualitative and quantitative assessment of the antimicrobial activity of the produced siderophore. To the best of authors’ knowledge, the present work is the first report focusing on a wide broad spectrum antimicrobial activity of a non-fluorescent siderophore and assaying its activity through a novel simple assay.

## Methods

### Bacterial strains and plasmids

A bacterial isolate, tentatively nominated as S33 and previously (Embaby et al. 2014a) isolated from agricultural soil contaminated with pear fire blight pathogen through an intensive program for isolation of antimicrobial activity producing bacteria, was used in this study as a siderophore producer. *Escherichia coli* JM109(DE3) (Promega Co. USA) was used in this study as a host recipient in transformation procedure for pSID/EGYII, a plasmid isolated from the bacterial isolate S33.

### Indicator strains

All indicator strains including human and plant pathogens along with their specifications used in this study were previously detailed mentioned (Embaby et al. 2014a). The indicator strain *Erwinia amylovora* strain EGY1 was deposited in DSMZ under the accession number: DSM 101800. Moreover, its 16S rDNA nucleotide sequence was previously deposited in the GenBank under the accession number: KM597069.1.

### Media

PYBG medium was used as the core siderophore production medium. It contained the following ingredients in % (w/v): 0.5 g peptone, 0.3 g beef, 0.5 g yeast extract, 0.5 g NaCl and 2 g glucose unless otherwise stated as reported previously (Embaby et al. 2014a). Pseudomonas base agar (LAB 108) plus X107 (CN: cetrimide and sodium nalidixate) supplement, modified King A, is used during the course of bacterial identification (King et al. 1954; Goto and Enomonto 1970). MYP medium (Mannitol Yolk Polymyxin) is used during carrying out biochemical profiling for the isolate of quest (Mossel et al. 1967; Fricker et al. 2008). Luria–Bertani (LB) (Sambrook et al. 1989) broth was used during the preparation of competent *E. coli* JM 109 (DE3) cells and the transformation procedure as well. However, all growth media encouraging the growth of the indicator strains were previously detailed described (Embaby et al. 2014a).

### Qualitative detection of siderophore

The nature of the antimicrobial agent existed in the cell free supernatant of the bacterial isolate S33 was investigated through three step procedure in the current study. The first test included boiling of the cell supernatant at 100 °C for 10 min. The second test involved incubation of the supernatant with protease K at 55 °C for 30 min. Whilst, the last test encompassed the incorporation of FeCl_3_ (at a final concentration of 100 µM) in the agar plate of *E. amylovora* strain EGY1 DSM 101800 prior employing this plate in the well cut diffusion test (mentioned below).

### Identification of the siderophore producing bacterial isolate

The siderophore producing bacterium (S33) was subjected to a detailed identification protocol including morphological, biochemical and molecular identification. Morphological and biochemical identification were carried out according to Bergey’s manual of determinative bacteriology (Bergeys 1990). However, molecular identification via 16S rDNA was performed according to a procedure previously reported (Eden et al. 1991) using previously described universal primers F8–27 (5′-AGAGTTTGATCCTGGCTCAG-3′) and R1510–1492 (5′-GGTTACCTTGTTACGACTT-3′) of *E. coli 16SrDNA* gene. The obtained nucleotide sequence was analyzed via BLASTN (Basic Local alignment Search Tool for nucleotides) algorithm of NCBI (National Center for Biotechnology Information) by aligning with 16S rDNA nucleotide sequences deposited in the international nucleotide databases (e.g., GenBank, EMBL, DDJB, etc.). Multiple sequence alignment (MSA) and phylogenetic tree construction were carried out to determine the taxonomic affiliation of the bacterial isolate S33 of quest using Geneious R9.0.4 software.

### Seed culture preparation

A single colony of the bacterial isolate S33, grown overnight on PYBG agar, was picked to inoculate 20 mL of PYBG broth. The inoculated broth was incubated for 18 h at 37 °C with agitation speed at 150 rpm (New Brunswick Incubator Shaker, USA) till achieving absorbance at 420 nm of bacterial growth 0.8.

### Inhibition spectrum and siderophore bioassay

Antimicrobial spectrum of the siderophore of quest was assessed using different indicator strains previously mentioned (Embaby et al. 2014a) as potential human and plant pathogens. Estimating the siderophore activity of the bacterial isolate S33 was carried out by well-cut diffusion test. Concisely, each indicator strain was activated overnight on its recommended medium on agar plate for an overnight at 37 °C. A single colony was picked to inoculate 20 mL of recommended broth in 100 mL Erlenmeyer flasks for each indicator strain. Each inoculated medium was incubated at agitation speed 150 rpm at 37 °C for overnight. After that, 1 mL (10^8^ CFU/ml) of this seed culture was taken to inoculate 100 mL of recommended agar medium for each indictor strain. The poured inoculated plates were allowed to solidify then wells were created in these plates using sterilized corkporer. One hundred micro-liters of cell free supernatant of overnight growing culture of the bacterial isolate S33 was added in each well in the inoculated plates with indicator strains separately. Then, these plates were incubated in upright position for overnight at 37 °C. Next day, zone of growth inhibition around each well was measured. The activity of the tested siderophore was expressed in siderophore arbitrary absolute units (SAU). One SAU is defined as inhibition zone diameter in mm produced by the tested siderophore on the bacterial indicator lawn. Here, the bacterial indicator lawn involved in assessment of siderophore activity in further experiments was *E. amylovora* strain EGY1 DSM 101800 unless otherwise stated. In addition, FeCl_3_ was incorporated only in the well cut diffusion agar plate of *E. amylovora* strain EGY1 DSM 101800 for qualitative detection purposes of the produced siderophore.

SAU/mL = inhibition zone diameter in mm. One thousand per volume of cell-free supernatant in μL.

### FeCl_3_ test

The effect of different concentrations of FeCl_3_ on siderophore production by the bacterial isolate S33 was evaluated. Briefly, 100 mL of PYBG broth in 250 mL Erlenmeyer flasks were inoculated with 2 mL of seed culture of the bacterial isolate S33 and were separately supplemented with different concentrations of FeCl_3_ (ranged from 0.0 to 20 µM). After incubation for an overnight with 150 rpm agitation speed at 37 °C, 1 mL of each culture was centrifuged (Mikro 200 Hettich, Germany) at 7000 rpm for 5 min. Cell free supernatants were collected separately and were stored at 4 °C till being tested for siderophore activity by well-cut diffusion assay as mentioned above using *E. amylovora* strain EGY1 DSM 101800 as an indicator strain.

### Siderophore typing

The siderophore of quest was typed as hydroxamate or catecholate type according to procedures reported previously (Arnow 1937; Dave and Dube 2000; Meyer et al. 1990). Tetrazolium test and Neilands spectrophotometric assay were carried out for detecting of hydroxamate siderophore type. However, Arnow’s test was employed to detect the catecholate siderophore type. In addition, the green yellow color of culture if any was checked both visibly and under ultra violet irradiation.

### Highlighting key determinants for siderophore production

Key determinants controlling siderophore production by the bacterial isolate S33 was addressed through a one-step simple procedure; Plackett–Burman experiment (Plackett and Burman 1946). In this experiment, nine factors namely (incubation time, glucose, initial pH of the production medium, incubation temperature, agitation speed, glycerol, starch, inoculum size and yeast extract) were tested to evaluate their impact on siderophore production. Each factor was tested in two levels; low level and high level coded as −1 and +1, respectively. Corresponding values for these coded values were given for each tested factor. A Plackett–Burman matrix with 20 trials was anticipated here (Table 1). Significant factors imposing influence on siderophore production was taken at P < 0.05. All trials were performed in triplicates using 50 mL of production medium in 250 mL Erlenmeyer flasks. Averages of three readings were considered. Minitab software 15.0 was used to create the matrix of Plackett–Burman design and to carry out the multiple linear regression as well. A polynomial equation (Eq. 1) from the first order was portrayed to explain the impact of the tested independent variables on the process outcome as follow:

**Table 1:** PBD showing levels of nine tested independent variables along with the response (SID EGY II activity)

Trial#	Independent variable									Dependent variable (Y) SID EGY II activity (SAU/mL)	
	X1	X2	X3	X4	X5	X6	X7	X8	X9	Exp	Pred
1	−1(24)	1(1)	1(7.2)	−1(25)	−1(120)	−1(0)	−1(0)	1(12)	−1(0.3)	280	327
2	1(48)	−1(0.5)	1(7.2)	1(28)	−1(120)	−1(0)	−1(0)	−1(10)	1(0.5)	330	353
3	1(48)	−1(0.5)	−1(6.2)	1(25)	−1(120)	1(1)	−1(0)	1(12)	−1(0.3)	290	277
4	1(48)	1(1)	1(7.2)	1(28)	−1(120)	−1(0)	1(1)	1(12)	−1(0.3)	250	209
5	−1(24)	−1(0.5)	1(7.2)	−1(25)	1(180)	−1(0)	1(1)	1(12)	1(0.5)	340	301
6	−1(24)	1(1)	1(7.2)	1(28)	1(180)	−1(0)	−1(0)	1(12)	1(0.5)	390	387
7	−1(24)	−1(0.5)	1(7.2)	1(28)	−1(120)	1(1)	1(1)	−1(10)	−1(0.3)	230	175
8	1(48)	1(1)	−1(6.2)	1(28)	1(180)	−1(0)	−1(0)	−1(10)	−1(.03)	340	327
9	−1(24)	1(1)	−1(7.2)	−1(25)	1(180)	1(1)	−1(0)	−1(10)	−1(0.3)	350	329
10	1(48)	1(1)	−1(6.2)	−1(25)	1(180)	1(1)	−1(0)	1(12)	1(0.5)	380	337
11	1(48)	1(1)	−1(6.2)	−1(25)	−1(120)	−1(0)	1(1)	−1(10)	1(0.5)	220	213
12	1(48)	−1(0.5)	1(7.2)	1(28)	1(180)	1(1)	−1(0)	−1(10)	1(0.5)	360	361
13	1(24)	−1(0.5)	−1(6.2)	−1(25)	1(180)	−1(0)	1(1)	−1(10)	1(0.5)	260	265
14	1(48)	−1(0.5)	1(7.2)	−1(25)	1(180)	1(1)	1(1)	1(12)	−1(0.3)	230	241
15	1(24)	1(1)	−1(6.2)	1(28)	−1(120)	1(1)	1(1)	1(12)	1(0.5)	190	167
16	1(48)	1(1)	1(7.2)	−1(25)	−1(120)	1(1)	1(1)	−1(10)	1(0.5)	160	207
17	−1(24)	−1(0.5)	−1(6.2)	−1(25)	−1(120)	−1(0)	−1(0)	−1(10)	−1(0.3)	320	303
18	1(48)	−1(0.5)	−1(6.2)	1(28)	1(180)	−1(0)	1(1)	1(12)	−1(0.3)	220	235
19	−1(24)	−1(0.5)	−1(6.2)	1(28)	−1(120)	1(1)	−1(0)	1(12)	1(0.5)	260	289
20	−1(24)	1(1)	−1(6.2)	1(28)	1(180)	1(1)	1(1)	−1(10)	−1(0.3)	130	177

X1: incubation time (h), X2: glucose % (w/v), X3: initial pH of production medium, X4: incubation temperature (°C), X5: agitation speed(rpm), X6: glycerol % (w/v)

X7: starch % (w/v), X8: inoculum size % (v/v) and X9: yeast extract % (w/v). Values between in the parenthesis indicate real values of independent variables

*Exp* experimental, *Pred* predicted

1 $$Y = \beta_{0} + \sum {Bixi}$$

### Antibiotic susceptibility test

Antibiotic sensitivity tests for the bacterial isolate S33 were performed. The sensitivity of the isolate of quest was tested against kanamycin, penicillin, ampicillin, chloramphenicol, tetracycline and tobramycin. A stock solution (100 µg/mL) from kanamycin and penicillin was prepared. After that, 100 µL of each antibiotic stock solution was separately added to 100 mL of PYB agar. The agar was poured in plates and after solidification, a fine touch of the bacterial isolate S33 was streaked on these plates. After 24 h incubation at 37 °C, all plates were checked for growth. For the remaining antibiotics (ampicillin, chloramphenicol, tetracycline and tobramycin), ready-made purchased discs containing these antibiotics at fixed concentrations (10 µg/disc) were used.

### Plasmid curing

The bacterial isolate S33 proving resistance towards all or some of the tested antibiotics mentioned above was subjected to a curing experiment according to a procedure previously reported by Chin et al. (2005) with slight modifications. Two curing agents; SDS and ethidium bromide were used in the present study. A stock solution from each curing agent was prepared; 10 mg/mL and 20 % of ethidium bromide and SDS, respectively was prepared. Ten milli-liters of PYBG broth in 100 mL Erlenmeyer flasks was mixed with 20 µL of each curing agent separately. Then, all flasks were inoculated with bacterial isolate S33 seed culture. After incubation for 18 h at 37 °C at 150 rpm, a subculture from each culture containing certain curing agent was prepared with newly addition of the curing agent at a fixed concentration. Sub culturing procedures were extended to 30 days. At time intervals (7, 14, 21 and 30 days), a loopful from each culture with a certain curing agent was used to inoculate a set of PYB agar plates incorporating the above mentioned antibiotics that the bacterial isolate S33 showed resistance against. Failure of a certain colony to grow on the agar plates is an indication about plasmid curing and vice versa.

### Plasmid isolation

Plasmid profiling for the bacterial isolate S33 was performed according to Sambrook et al. (1989). Integrity and quality of the isolated plasmid was checked by running on 1 % agarose gel electrophoresis and further visualization under ultra violet (UV) using UV-Transillumuator. Then, the isolated plasmid was kept at −20 °C till being used in transformation procedures.

### Transformation of *E. coli* JM 109 (DE3)

Chemical competent cells of *E. coli* JM 109 (DE3) were prepared according to a procedure reported previously by Sambrook et al. (1989). Then, the plasmid isolated from the bacterial isolate S33 was used to transform the competent cells of *E. coli* JM 109 (DE3) according to a procedure reported previously (Sambrook et al. 1989).

### Testing siderophore activity in *E. coli* JM 109 (DE3) transformants

Transformants of *E. coli* JM109(DE3), picked on LB plates containing an appropriate concentration of an antibiotic where the bacterial isolate S33 showed resistance against, were tested to prove acquiring of siderophore trait or not. Briefly, the transformants were grown overnight at 150 rpm in 100 mL LB broth plus an appropriate concentration of the same antibiotic (the selectable marker previously used during transformants’ picking) in 250 mL Erlenmeyer flasks. Next day, cells were harvested via centrifugation at 7000 rpm for 5 min. Breakage of cell pellets was carried out via sonication at 14,000 Hz for 5 min through 5cycles discontinuously on ice with a time interval of 1 min between the cycles. Then, cell debris was harvested via centrifugation at 7000 rpm for 5 min. The cell free supernatant was kept at −20 °C till being used in assessment of siderophore activity in further experiments through well-cut diffusion test as mentioned above using *E. amylovora* strain EGY1 DSM 101800 as an indicator strain.

## Results

### *Pseudomonas aeruginosa* strain EGYII DSM 101801

**Fig. 1:**
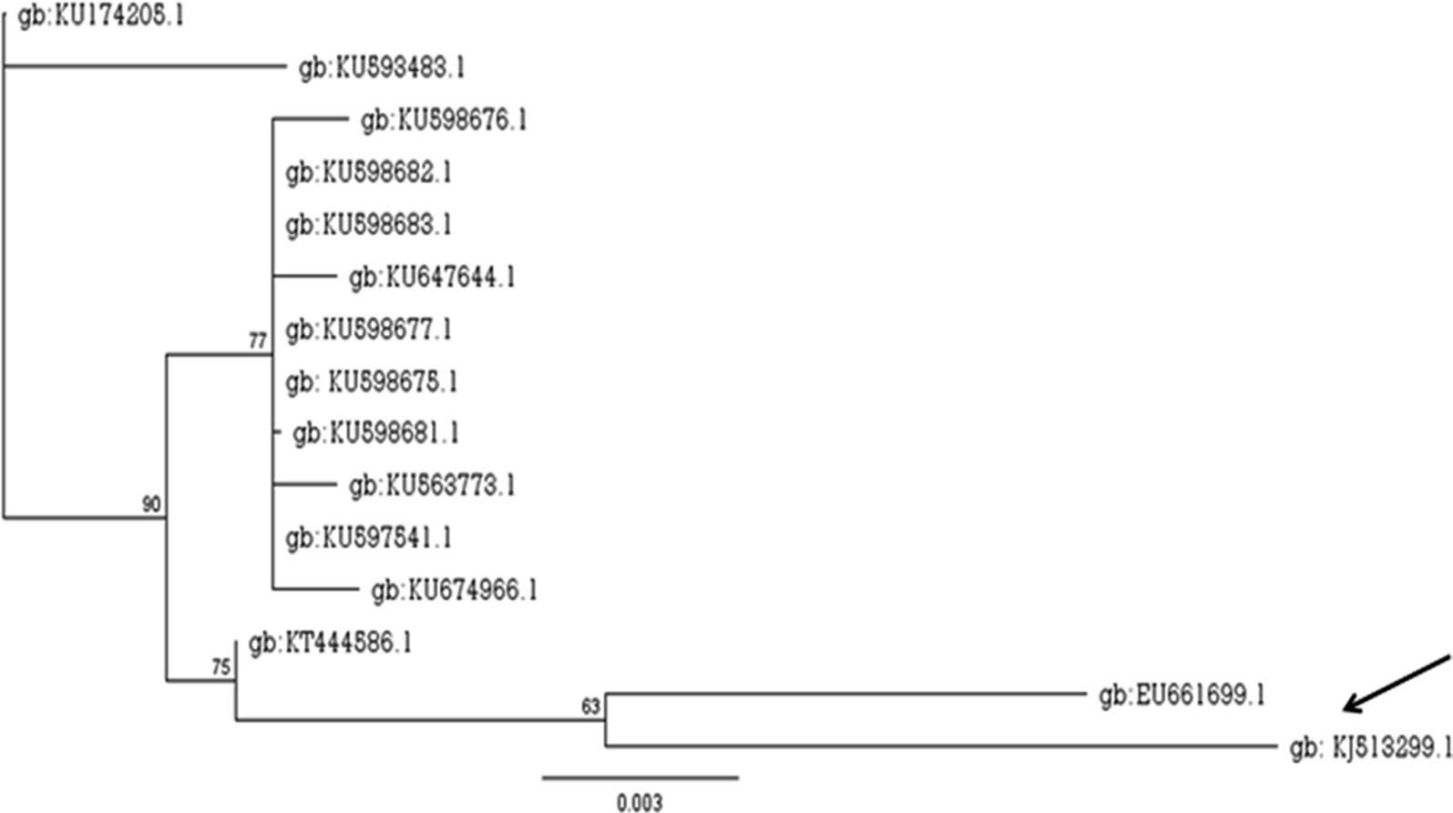
Neighbor-joining tree showing the phylogenetic relationship between 16S rDNA sequence of the bacterial isolate S33 (taxonomically designated as *Pseudomon*as *aeruginosa*) and other 16S rDNA sequences belonging to closely related bacteria. Hits are expressed by their accession numbers in international nucleotide databases. *Arrow* indicates *P.* *aeruginosa* strain EGYII. Phylogenetic tree was constructed via Geneious R9.0.4 software. *Numbers on branch nodes* represent bootstrap values (100 re-samplings)

An antimicrobial agent producing-bacterium, previously isolated from an agricultural soil and nominated as S33, was identified in this study through a three step procedure. Morphological features and biochemical profiling of the isolate of quest were demonstrated in Table 2. Morphological features revealed that this isolate is Gram-negative, rods and its colonies have given yellowish green pyoverdine fluorescence (fluorescein) under UV upon its growth on Pseudomonas base agar (modified King A medium) supplemented with CN. Whilst, the biochemical profile conferred that the isolate is oxidase positive, grow at 42 °C, β-hemolytic on blood agar; important criteria for *P. aeruginosa*. After DNA sequencing, 753 nucleotides of 16S rDNA sequence were obtained. BALSTN analysis, MSA along with phylogenetic tree (Fig. 1) verified that the 16S rDNA nucleotide sequence of quest is closely related (with 98 % similarity) to a bacterium called *P. aeruginosa* strain NBRAJG78 (its 16S rDNA nucleotide sequence deposited in the GenBank under the accession number (gb: EU661699.1). Consequently, morphological, biochemical and molecular identifications inferred that the bacterial isolate S33 of quest is taxonomically affiliated to *P. aeruginosa*. Further, it was designated as *P. aeruginosa* strain EGYII and its 16S rDNA nucleotide sequence was deposited in the GenBank under the accession number (gb: KJ513299.1). Moreover, this strain was deposited in DSMZ and was assigned an accession number (DSM: 101801).

**Table 2:** Morphological, biochemical profile and antibiotic sensitivity tests of the bacterial isolate S33

Test	Result
Cell shape	Rods
Gram stain	−ve
Spore formation	−ve
Growth on MYP	Non-mannitol utilizer
Motility	−ve
Fluorescence on Pseudomonas base agar (modified King A)	Yellowish green pyoverdin fluorescence under UV (fluorescein)
Growth at pH 6–8	+ve
Growth at 42 °C	+ve
Growth at 7.5 % NaCl	−ve
Starch hydrolysis	−ve
Casein hydrolysis	+ve
Tween 20 hydrolysis	−ve
Nitrate reduction	+ve
Catalase	+ve
Oxidase	+ve
Urease	+ve
Indole production	−ve
Citrate utilization	+ve
Voges–Proskauer (VP)	−ve
Methyl Red (MR)	+ve
Gelatin liquefaction	−ve
Hemolysis on sheep blood agar	+ve β-hemolytic
Glucose fermentation	+ve (yellow/blue: O/NF)
Galactose fermentation	+ve (yellow)
Sucrose fermentation	ND
Arginine utilization	−ve
Antibiotic sensitivity	
Kanamycin	Sensitive
Penicillin	Resistant
Ampicillin	Resistant
Chloramphenicol	Sensitive
TOB10	Sensitive
Tetracycline	Resistant

*O*/*NF* oxidative/non-fermentative, *ND* not determined resistant

*−ve* negative result, *+ve* positive result

The antibiotic profile of *P. aeruginosa* strain EGYII DSM 101801 was evaluated concerning some antibiotics mentioned in the section of materials and methods. The strain showed resistance towards penicillin, ampicillin and tetracycline (Table 2). However, the test strain exhibited sensitivity towards chloramphenicol, kanamycin and tobramycin (Table 2).

### Verifying the siderophore nature of SID EGYII

A three step approach was employed to unravel the nature of the substantial antimicrobial agent produced by *P. aeruginosa* strain EGYII DSM 101801. Firstly, the antimicrobial activity was retained 100 % after boiling of the crude supernatant at 100 **°C** for 10 min. In addition, the antimicrobial agent was not adversely affected by incubation with proteinase K for 30 min at 55 **°C**. This in turn indicated the non-proteinous (most probably non-bacteriocin) nature of the antimicrobial agent of quest. Secondly, no zone of inhibition was detected in well-cut diffusion assay of *E. amylovora* strain EGY1 DSM 101800 containing 100 µM FeCl_3_; however, agar diffusion plates prepared without FeCl_3_ showed perceivable zones of inhibition around the wells containing the antimicrobial agent of quest. This verified the siderophore nature of the tested antimicrobial agent.

**Fig. 2:**
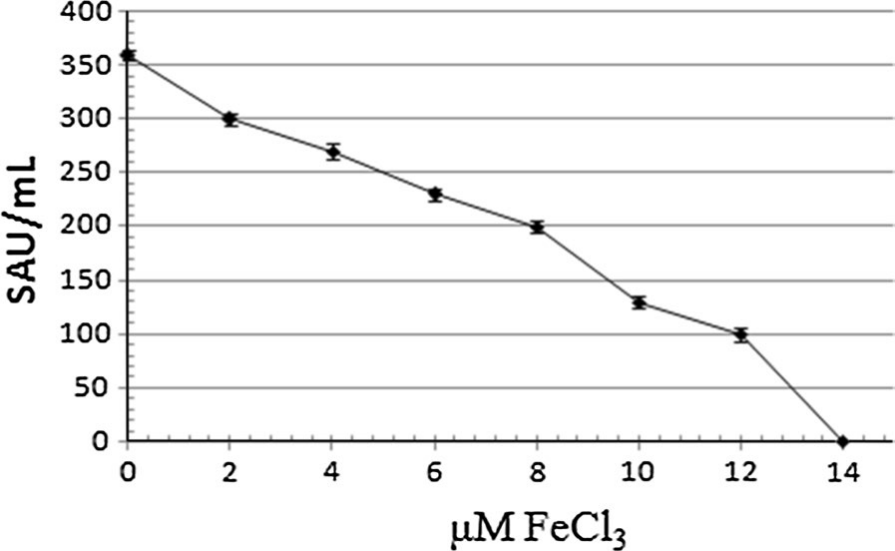
Effect of FeCl_3_ concentrations on siderophore production by *P. aeruginosa* strain EGYII DSM 101801. Values are averages of three readings ± standard error bars

Thirdly, the effect of adding different concentrations of FeCl_3_ on the production of the antimicrobial agent was assessed. Figure 2 showed that the highest amount of the antimicrobial agent (350 AU/mL) was achieved at zero concentration of FeCl_3_ in the production medium. Moreover, increasing the concentration of FeCl_3_ in the production medium gradually from zero to 12 µM resulted in alleviation in the amount of the produced antimicrobial agent (100 AU/mL). However, addition of concentrations of FeCl_3_ beyond 12 µM (threshold level) would result in complete repression in the production of the antimicrobial agent of quest. Conclusively, these results strongly verified the siderophore nature of the antimicrobial agent of quest. This antimicrobial activity included in the supernatant of *P. aeruginosa* strain EGYII DSM 101801 obtained upon its growth in PYBG broth after 24 h was nominated as SID EGYII.

### Partial characterization of crude SID EGYII

**Fig. 3:**
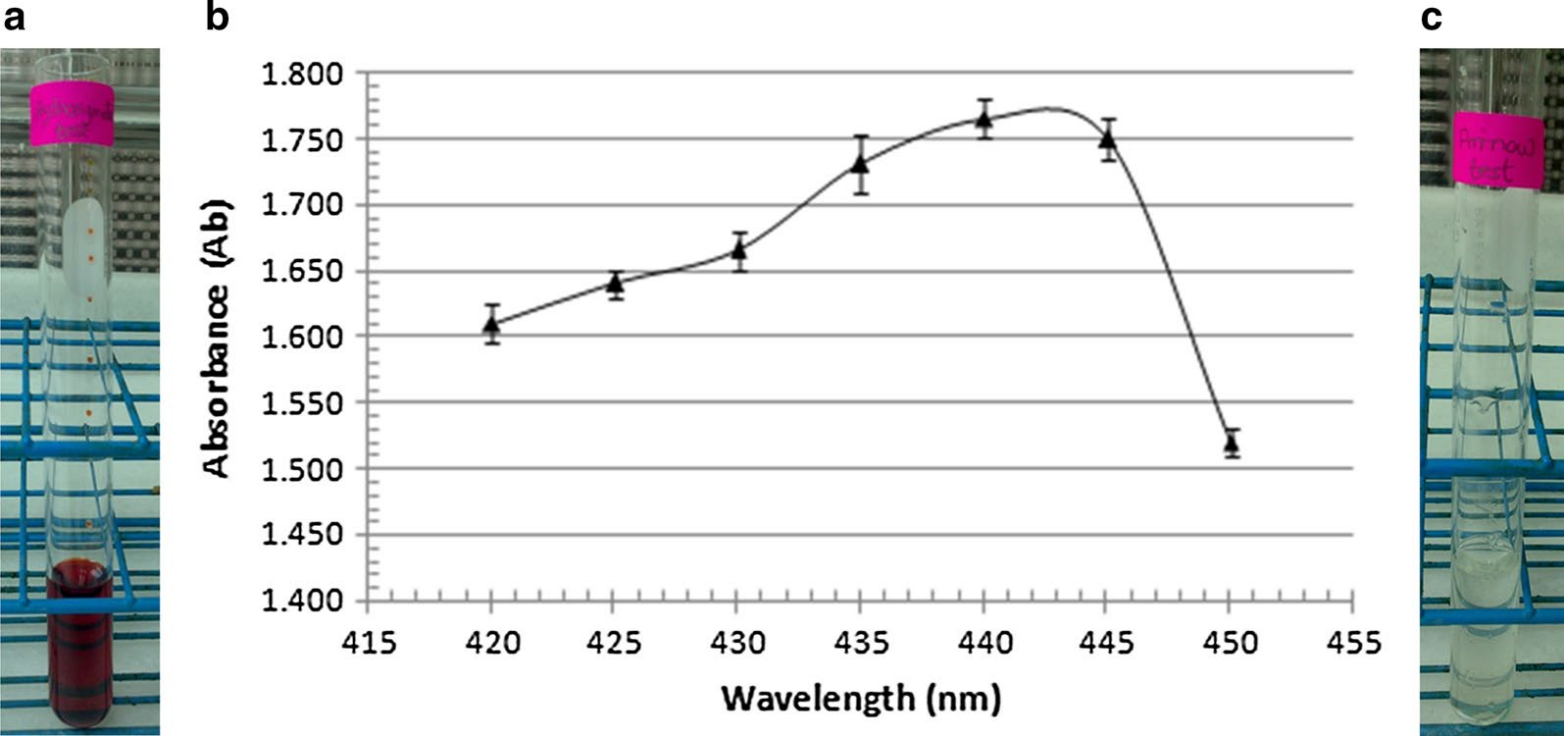
SID EGYII typing. **a** Tetrazolium test showing a *red brown color* of ferrate hydroxamate SID EGYII. **b** Neilands spectrophotometric assay showing a peak of absorbance between 420 and 450 nm of hydroxamate SID EGYII. Values are averages of three readings ± standard error bars. **c** Arnow’s test showing absence of *red color* (no catecholate siderophore)

To have more insights about the type of SID EGYII nature, both hydroxamate and catecholate tests were carried out. Present data revealed the hydroxamate nature of the siderophore of quest. This was evinced from deep red color formation upon addition of tetrazolium salt (Fig. 3a). Moreover, the noticeable peak obtained between 420 and 450 nm in Neilands spectrophotometric assay gave additional evidence about the hydroxamate nature of SID EGYII due to the formation of ferrate hydroxamate complex (Fig. 3b). While, negative results from Arnow’s test conferred the absence of catecholate nature of SID EGYII (Fig. 3c).

Moreover, neither green nor blue yellowish fluorescence of *P. aeruginosa* strain EGYII DSM 101801 culture was noticeably detected even after exposure to ultra-violet illumination. This implies that SID EGYII has a non-fluorescent nature.

Results of antimicrobial spectrum of the tested SID EGYII revealed that it has a wide spectrum antimicrobial activity against some of the tested Gram-negative and Gram-positive human and plant pathogens (Table 3). The growth of *Salmonella paratyphi*, *Shigella boydii*, *Enterobacter* sp., *Enterococcus faecalis*, *E. coli*, *Vibrio parahaemolyticus*, *E. amylovora* strain EGY1 DSM 101800, *Bacillus subtilis* and *Staphylococcus aureus* was adversely affected by SID EGYII. Interestingly, SID EGYII displayed antimicrobial activity against two species of *Candida*, two potential human pathogens, *Candida albicans* and *C. tropicalis*. However, the other tested indictor strains did not being affected by SID EGYII.

**Table 3:** Sensitivity of some indicator strains to SID EGY II

Indicator strain	SID EGY II activity (SAU/mL: inhibition zone diameter in mm)^a^	Gram-nature
Bacteria		
*Acinetobacter*sp. (clinical isolate, Faculty of Medicine, University of Alexandria)	0.00	Gram-negative
*Bacillus subtilis* NCTC 10400/ATCC 6633	0.00	Gram-positive
*B. subtilis* (environmental strain isolated from soil, Egypt)	173.3 ± 6.7	Gram-positive
*B. subtilis* AS1 (environmental strain isolated from soil, Egypt)	0.00	Gram-positive
*Bacillus subtilis* EMBLAKE (environmental strain isolated fromEl-Mahmoudia lake, Egypt)	0.00	Gram-positive
*Bacillus* sp. YAS 1(environmental strain isolated from soil, Egypt)	0.00	Gram-positive
*Bacillus* sp. Ash2 (environmental strain isolated from soil, Egypt)	0.00	Gram-positive
*B. cereus* (environmental strain isolated from soil, Egypt)	0.00	Gram-positive
*B. licheniformis* SHG2 (environmental strain isolated from soil, Egypt)	0.00	Gram-positive
*B. licheniformis* SHG6(environmental strain isolated from soil, Egypt)	0.00	Gram-positive
*B. licheniformis* SHG10 DSM 28096 (environmental strain isolated from soil, Egypt)	0.00	Gram-positive
*Campylobacter jejuni* NCTC 11322/ATCC 29428	0.00	Gram-negative
*Citrobacter* sp. (clinical isolate, Faculty of Medicine, University of Alexandria, Egypt)	0.00	Gram-negative
*Clostridium perfringens* NCTC 8237/ATCC 13124	0.00	Gram-positive
*Escherichia coli* NCTC 12241/ATCC 25922	250 ± (5.7)	Gram-negative
*E. coli* (clinical isolate, Faculty of Medicine, University of Alexandria, Egypt)	0.00	Gram-negative
*Enterococcus* sp.(clinical isolate, Faculty of Medicine, University of Alexandria, Egypt)	0.00	Gram-negative
*Enterobacter aerogenes* NCTC10006/ATCC13043	0.00	Gram-negative
*Enterobacter* sp. (clinical isolate, Faculty of Medicine, University of Alexandria)	130 ± (9.1)	Gram-negative
*Enterococcus faecalis* NCTC 12697/ATCC 29212	336 ± (11.2)	Gram-negative
*E. amylovora* strain EGY1 DSM 101800 (Plant pathology department, Faculty of Agriculture, University of Alexandria, Egypt)	390 ± (5.8)	Gram-negative
*Klebsiella* sp. (clinical isolate, Faculty of Medicine, University of Alexandria, Egypt)	0.00	Gram-negative
*Listeria innocua* NCTC 11288/ATCC 33090	0.00	Gram-negative
*Proteus* sp. (clinical isolate, Faculty of Medicine, University of Alexandria, Egypt)	0.00	Gram-negative
*Pseudomonas aueroginosa* (clinical isolate, Faculty of Medicine, University of Alexandria, Egypt)	0.00	Gram-negative
*Staphylococcus aureus* NCTC 12981/ATCC 25923	176 ± (6.3)	Gram-positive
*S. aureus* (clinical isolate, Faculty of Medicine, University of Alexandria, Egypt)	0.00	Gram-positive
*S. epidermidis* NCTC 13360/ATCC 12228	0.00	Gram-positive
*Shigella boydii* ATCC 9207	110 ± (5.8)	Gram-negative
*Salmonella typhi* (clinical isolate, Faculty of Medicine, University of Alexandria, Egypt)	0.00	Gram-negative
*S. paratyphi* (clinical isolate, Faculty of Medicine, University of Alexandria,Egypt)	236 ± (8.8)	Gram-negative
*S. typhimurium* NCTC 12023/ATCC *14028*	0.00	Gram-negative
*Saccharomyces cerevisiae* NCPF 3178	0.00	–
*Vibrio parahaemolyticus* NCTC 3178	160 ± (11.6)	Gram-negative
Fungi and yeasts		
*Aspergillus brasiliensis* NCPF2275/ATCC16404	0.00	–
*Candida albicans NCPF* 3179/ATCC10231	143 ± (8.6)	–
*Candida tropicalis* (human clinical isolate from diabetic foot, Department of Microbiology, Medical Research Institute, University of Alexandria, Egypt)	126.6 ± 3.34	**–**

^a^Mean of three readings

Values in between the parenthesis indicate standard error (SE)

### Production of SID EGYII by *P. aeruginosa* strain EGYII DSM 101801

The influence of nine physicochemical determinants on SID EGYII form *P. aeruginosa* strain EGYII DSM 101801was tested through Plackett–Burman design. Table 1 displayed experimental vs. predicted values obtained upon carrying out the twenty trials of the employed matrix. Results indicated that the arbitrary units of SID EGYII ranged from 130 to 390 SAU/mL. This in turn addressed the indispensable need to carry out screening experiment in order to look for the key determinants controlling the process output. Multiple linear regression of experimental data revealed that only two out of nine factors imposed significant influence (P < 0.05) on the process outcome (Table 4). These two factors are starch and agitation speed. All factors that did not impose significance consequence on the process outcome were either omitted (glucose and glycerol) or were settled at their lowest coded values (incubation temperature, incubation time, initial pH of the production medium, inoculum size and yeast extract) in further experiments. Further, starch was omitted from the production medium because it was negatively impacted the process output. Whilst, agitation speed that had positive significant consequence on SID EGY II production was used in further experiments at its highest coded value (180 rpm). Hence, the newly tailored SID EGYII production medium was PYB containing the same concentrations of ingredients included in PYBG except that glucose was omitted and yeast extract concentration was 0.3 % (w/v). The other physical factors were set at pH (6.2), incubation temperature (28 **°C**), inoculum size [10 % (v/v)], agitation speed (180 rpm) and incubation time (24 h). A polynomial equation (Eq. 2) from the first order in terms of coded values was established to explore the linear relation between the independent variables and the process output as follow:

**Table 4:** Regression summary for full polynomial equation for evaluation of siderophore production using PBD

Independent variable	Coefficient symbol	*Β* coefficient	P value	t value	% confidence
Intercept	B0	274	4.7e−11	29.46	99.99
Incubation time (X_1_, hours)	B1	2.0	0.8300	0.22	–
Glucose (X_2_, w/v %)	B2	−6	0.5300	−0.65	–
pH (X_3_)	B3	15	0.1400	1.6	–
Incubation temperature (X_4_, °C)	B4	−6	0.5300	−0.65	–
Agitation speed (X_5_, rpm)	B5	22	0.0390	2.37	96.1
Glycerol (X_6_, w/v %)	B6	−18	0.0800	−1.94	–
Starch (X_7_, w/v %)	B7	−55	0.0002	−5.9	99.98
Inoculum size (X_8_, v/v %)	B8	3	0.7500	0.32	–
Yeast extract (X_9_, g/L)	B9	14	0.1600	1.51	–

* Significant P < 0.05 and R^2^ = 0.83 and Adjusted R^2^ = 0.68 and P value for the model = 0.007

2 $$Y = 274 + 2X1 - 6X2 + 15X3 - 6X4 + 22X5 - 18X6 - 55X7 + 3X8 + 14X9$$

**Fig. 4:**
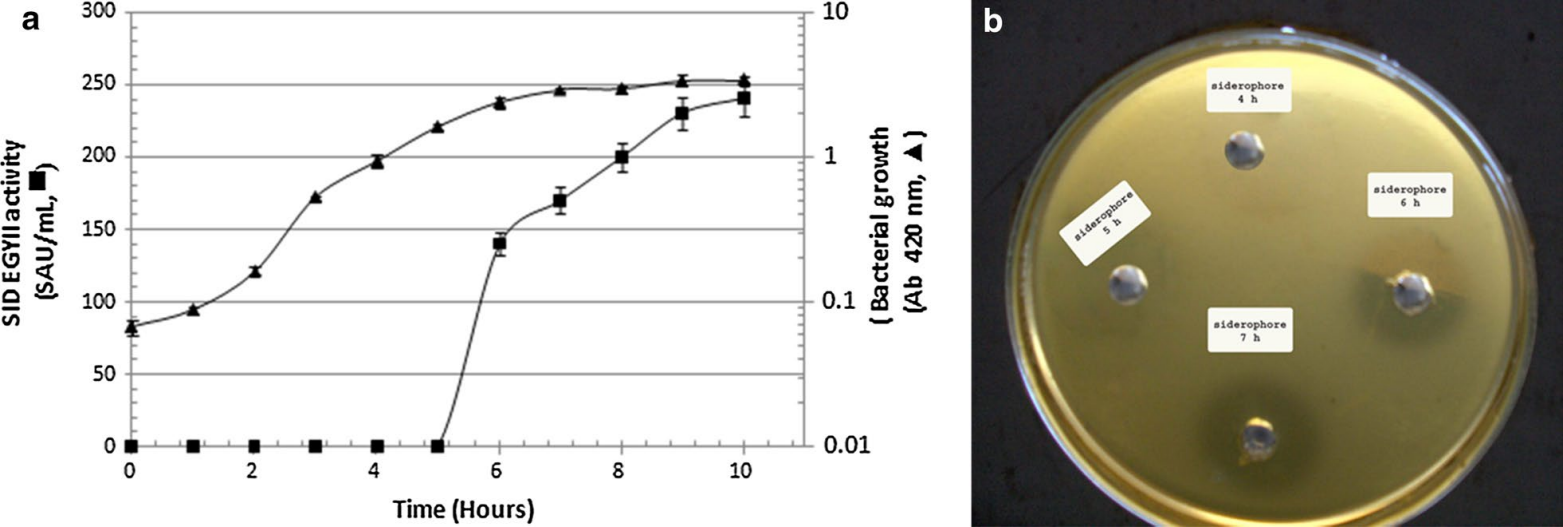
**a** Time line production of SID EGYII during the growth of *P. aeruginosa* strain EGYII DSM 101801 on PYB medium. Values are averages of three readings ± standard error bars. **b** Well-cut diffusion assay using the indicator strain *E. amylovora* strain EGY1 DSM 101800 showing the onset of SID EGYII production after 6 h of the growth of the producer bacterial strain on PYB medium

The onset of SID EGYII production throughout the course of *P. aeruginosa* strain EGYII DSM 101801 growth on modified PYB medium was monitored. The first onset of SID EGYII (140 SAU/mL) was noticeable after 6 h of the bacterial growth (i.e., very late exponential phase or onset of the stationary phase) (Fig. 4a, b). Increased levels of SID EGYII were observed during the stationary phase of bacterial growth.

### SID EGYII activity and pSID/EGYII plasmid

**Fig. 5:**
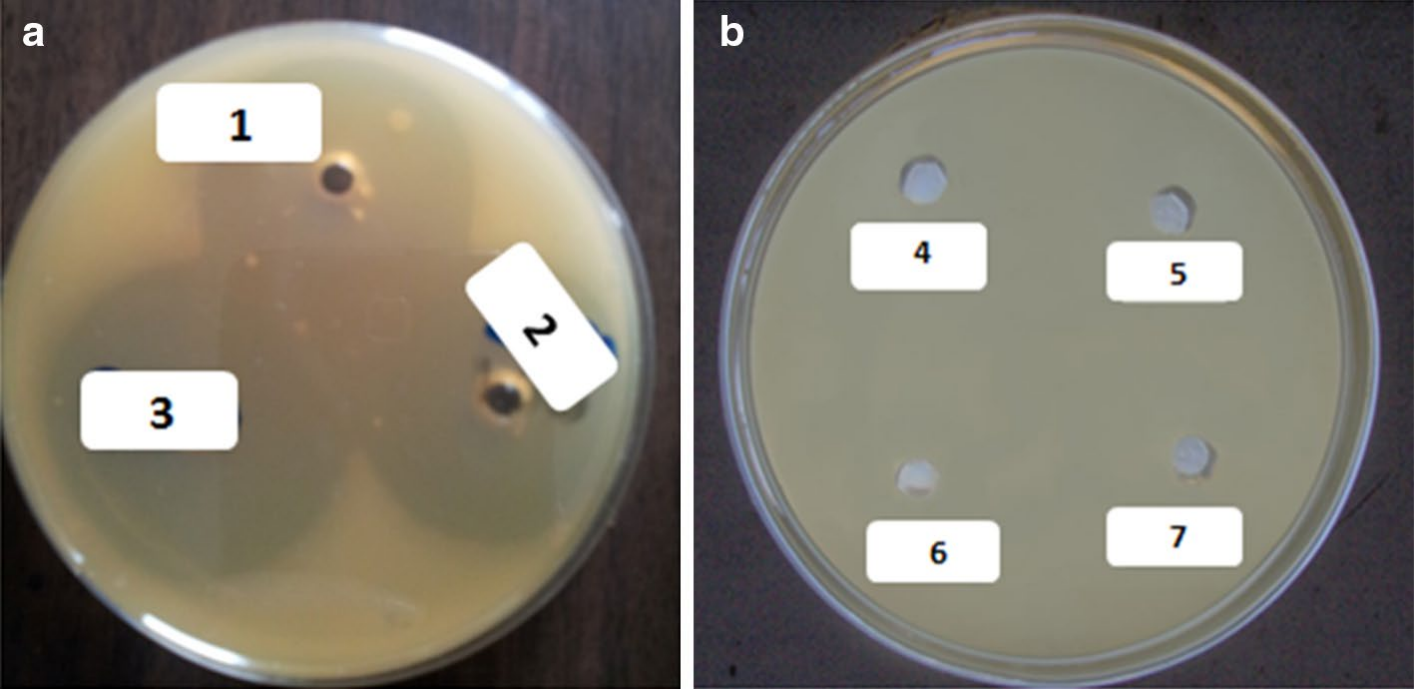
**a** Cell lysate of *E. coli* JM109 (DE3) pSID/EGYII transformants (three clones: *1*, *2* and *3*) showing activity of SID EGY II against the indicator strain *E. amylovora* strain EGY1 DSM 101800. **b** Cell lysate of *E. coli* JM109 (DE3) host (four replica: *4*, *5*, *6* and *7*) showing no activity against the indicator strain *E. amylovora* strain EGY1 DSM 101800

Trials for plasmid curing failed along 30 days monitoring at fixed time intervals previously mentioned in the section of materials and methods. Cells *P. aeruginosa* strain EGYII DSM 101801 could grow well in plates containing penicillin, ampicillin and tetracycline, separately after exposure to the curing agents (SDS and ethidium bromide). Plasmid profile of *P. aeruginosa* strain EGYII DSM 101801 revealed the presence of either one type of plasmid with different conformational forms corresponding to different migration rates on agarose gel or more than one plasmid with different molecular weights. This total plasmid mixture was designated as pSID/EGYII. Regardless the nature of this plasmid mixture, it was totally used to transform competent *E. coli* JM109(DE3) cells. Transformants were picked from LB plates containing ampicillin at a final concentration of 10 µg/mL as a selectable marker. The tranasformants were nominated as *E. coli* JM 109(DE3) pSID/EGYII. To verify that the gene encoding siderophore trait is harbored on pSID/EGYII or not, these transformants were tested against *E. amylovora* strain EGY1 DSM 101800 using well-cut diffusion test. Data conferred that the cell lysate of the transformant *E. coli* JM 109 (DE3) pSID/EGYII did succeed to inhibit the growth of *E. amylovora* strain EGYI DSM 101800 (Fig. 5a) however, the host cells *E. coli* JM 109 (DE3) did not (Fig. 5b). That is to confirm that the genes encoding for siderophore trait in the wild type *P. aeruginosa* strain EGYII DSM 101801 are carried on the plasmid (pSID/EGYII) not on the bacterial chromosome.

## Discussion

It was reported that the majority of siderophores produced by fluorescent Pseudomonads (e.g., *P. aeruginosa*, *P. flurescens*, *P. putida*, *P. chlororaphis* and *P. syringae*) are fluorescent siderophores mainly belonging to pyoverdines group (Albesa et al. 1985; Henry et al. 1991; Meyer 2000; Meyer et al. 2002; Visca et al. 2007; Unni et al. 2014). The up to date review of literature contains a plethora of reports discussing some issues of pyoverdines and pyochelins, yellowish green and yellowish blue fluorescent siderophores, respectively from *P. aeruginosa*, concerning nature, structure, production and characterization of these siderophores (Meyer et al. 1997; Cox and Adams 1985). On the contrary, a few number of reports highlighted the nature of non-fluorescent siderophores from fluorescent and non-fluorescent Pseudomonads (Bultreys et al. 2001; Mehri et al. 2011; Unni et al. 2014). In this context, the present research underlines a non-fluorescent siderophore from a new environmental strain of *P. aeruginosa*. Reportedly, Chromazurol-Shuttle assay is an universal assay for qualitative and quantitative assessment of siderophore activity (Schwayn and Neilands 1987). However, its high cost along with tedious cumbersome procedure encountered in its preparation would greatly restrict its wide use in siderophore studies.

In the light of this background, a previously isolated antimicrobial agent-producing soil bacterium (S33), was identified in this study as *P. aeruginosa* strain EGYII DSM 101801 via a two-step identification procedure: biochemical identification and molecular identification. Previously, this antimicrobial agent was proved to antagonize the growth of *E. amylovora* strain EGY1 DSM 101800, a fire blight plant pathogen, through the well-cut diffusion assay (Embaby et al. 2014a). Herein, a simplified low cost effective three step approach was employed to unravel the nature of the antimicrobial agent of quest in order to prove whether it is a siderophore or non-siderophore. Unlike reported heat stable antimicrobial peptides designated as bacteriocins (Kayalvizhi and Gunasekaran 2008; Banerjee et al. 2013; Embaby et al. 2014a), the tested antimicrobial agent retained its full activity after boiling for 15 min and even after incubation with proteinase K for 30 min at 55 °C. Clearly, these two findings underpin the non-proteinous nature (i.e., non-bacteriocin nature) of the tested antimicrobial agent. Further, absence of zone of inhibition in *E. amylovora* strain EGYI DSM 101800 well-cut diffusion assay in presence of 100 µM FeCl_3_ indirectly emphasized that the antimicrobial agent of quest is a siderophore in nature. This suppressed or masked antimicrobial activity of SID EGYII against *E. amylovora* strain EGYI DSM 101800 could be attributed to the presence of FeCl_3_ in the growth medium of *E. amylovora* strain EGYI DSM 101800 in an excess concentration satisfying the bacterial iron requirements. As a consequence, the siderophore activity retained in the cell free supernatant of *P. aeruginosa* strain EGYII DSM 101801 was nominated as SID EGYII. The present simple qualitative assay using FeCl_3_ in well-cut diffusion assay is a cheap efficient alternate to the currently universal applied assay; Chromazurol-Shuttle assay. The latter is not only an expensive assay but also a multistep cumbersome assay. Concomitantly, quantitative assessment of SID EGYII activity by expressing the arbitrary units (SAU) taking into account the diameter of inhibition zone around the test strain is a good alternate to Chromazurol-Shuttle assay. Present finding reveals that SID EGY II shows 350 SAU/mL upon cultivating of *P. aeruginosa* strain EGYII DSM 101801 in PYB medium. Despite the advantages of the newly qualitative and quantitative assay presented here, it bears one limitation. It is a semi universal assay as long as various siderophores of numerous microbial origins would likely display different spectra of antimicrobial activity against various test bacterial strains. In other words, the activity of each newly antimicrobial agent has to be tested qualitatively using the well-cut diffusion assay incorporating FeCl_3_ to prove its siderophore nature; however, selecting the target test bacterial strain is not an easy task.

Further evidences about the siderophore nature of SID EGYII were obtained by tracing its activity after 24 h of *P. aeruginosa* strain EGYII DSM 101801 growth in PYBG broth in presence of various concentrations of FeCl_3_ (0–20 µM). A marked decrease in the antimicrobial activity of SID EGYII was noticeable by increasing the concentration of FeCl_3_. Siderophores biosynthesis might be possibly repressed by increasing the outside concentrations of FeCl_3_ in the growth medium. As the cells would intend to save its energy included in the pathway of siderophores’ biosynthesis. Particularly, siderophores biosynthesis is most probably a multiple gene cassette dependent pathway not a single gene dependent pathway. Likewise present findings, other previously reported findings stated that siderophore production is inversely proportional with the amount of FeCl_3_ incorporated in the medium during the growth of the producer strain with different threshold levels of Fe^3+^ repression siderophoregenesis (Albesa et al. 1985; Budzikiewicz 1993; Bholay et al. 2012; Gaonkar and Bhosle 2013; Sreedevi et al. 2014). Unlike other finding of Calugay et al. (2003) stated that siderophoregenesis from *Magnetospirillum magneticum* AMB-1 was stimulated by iron sufficiency with at least an initial Fe^3+^ concentration of 6 µM rather than iron starvation.

No fluorescence could be detected in the crude cell free supernatant containing SID EGYII either by naked eye or even after exposure to ultra-violet illumination. This verified the non-fluorescent nature of SID EGYII. Unlike pyoverdines and pyochelins; two fluorescent siderophores produced by *P. aeruginosa* (Meyer 2000), *P. cepacia* (Sokol et al. 1992) and *P. flurescens* (Xiao and Kisaalita 1995), SID EGYII is a non-fluorescent siderophore. Present data underpins the production of unusual siderophore (SID EGY II) by *P. aeruginosa* strain EGYII DSM 101801. Typically, majority of siderophores reported till now are sorted in three major classes based on the functional group they utilize to chelate irons: catecholate, hydroxamate and mixed ones (Crichton and Charloteaux-Wauters 1987; Winkelmann 1991; Crosa and Walsh 2002; Calugay et al. 2003; Pluháček et al. 2016). Likewise some siderophores of different bacterial origins, SID EGYII proved to exert hydroxamate nature. Present results address the indispensable need to thoroughly study the chemical structure of this unusual siderophore in a prospective work.

As a rule of thumb, the more wide spectrum an antimicrobial agent would show, the high potential this antimicrobial agent would have. In this context, SID EGYII antimicrobial activity was extensively studied against various microbial pathogens. Interestingly, SID EGYII displayed a wide spectrum antimicrobial agent against some human and plant pathogens belonging to Gram-negative *(Salmonella paratyphi*, *Shigella boydii*, *Enterobacter* sp., *Enterobacter faecalis*, *E. coli*, *V. parahaemolyticus* and *Erwinia amylovora* strain EGYI DSM 101800) and Gram-positive bacteria (*B. subtilis* and *S. aureus*) and some pathogenic yeasts (*C. albicans* and *C. tropicalis*) as well. There exist discrepancies among responses of different tested bacterial members of different species/genera and even of same species towards SID EGYII. These discrepancies might be attributed possibly to the presence of siderophores’ receptors on the cell membranes of bacterial members that did not affect adversely by SID EGYII and their absence in bacterial members that did affect adversely by SID EGYII activity. These siderophore’s membrane receptors would help the bacteria to satisfy their iron nutritional requirements.

So far, a little attention has been paid to the antimicrobial activity of the siderophores themselves. For instance, Siderophore of the yeast *Aureobasidium pullulans* displayed potential antimicrobial activities against *Vibrio anguillarum* and *V. parahaemolyticus*; two pathogenic bacteria isolated from diseased animals (Wang et al. 2009). A recent study highlighted the wide spectrum antimicrobial activity of a siderophore, produced by Endophytic Fungus *Acremonium sclerotigenum*, against *B. cereus*, *B. subtilis*, *S. aureus* and *P. aeruginosa* (Prathyusha et al. 2015). On the contrary, much researches have been devoted for using siderophores as antibiotic conjugates complex to achieve better drug delivery systems in medical applications (Page 2013). Taking into account that there exists a little bit antimicrobial activity associated with the siderophore component of these complexes when being used as carriers. Duration of the potential antimicrobial activity encountered in the tested siderophore is a crucial issue. In this regard, SID EGYII exhibited a long last antimicrobial activity till 48 h and probably beyond this time. This in turn gains SID EGYII a privilege over deferoxamine that was reported to display antimicrobial activity against *S. aureus* after repeated additions of deferoxamine in the growth medium after 24 h (Hartzen et al. 1989). Hence, the wide spectrum antimicrobial activity of SID EGYII alongside its long lasting antimicrobial activity might indicate the high potential of SID EGYII to target certain pathogens that have implicated in human and plant pathogenesis; particularly those displaying multidrug resistances.

Normally, monitoring the expression profile of a gene encoding of an industrially important product of microbial origin is a subject of a prime importance in the agenda of commercialized bio-processing. This addressed the indispensable need to trace the expression of SID EGYII activity throughout the growth course of *P. aeruginosa* strain EGYII DSM 101801 in PYB broth (siderophore production medium). Late expression of genes encoding SID EGY II activity was noted nearly at the end of exponential phase; whilst, maximum SID EGYII activity was achieved during the stationary phase. Generally, the onset of expression for a gene encoding a certain trait among bacterial candidates in bio-processing varies widely among different bacterial members belonging to different genera and species and even among different strains belonging to the same species and subspecies. Present finding is partially in accordance with others who reported that perceivable levels of siderophores were successfully traced after 5 h of incubation of *P. fluorescens*, *P. aeruginosa*, *P. putida* and *P. mosselii,* separately in casamino acid (CCA) medium and succinate medium (SM) followed by an elevation in the level of the produced siderophores from 24 to 48 h (Ines et al. 2012). In addition, siderophore production from *P. fluorescens* was reported to be initiated after 6 h of bacterial growth achieving maximum production after 24 h (Tailor and Joshi 2012). However, siderophore production from *P. aeruginosa* BUP2 occurred during the late senescence phase (96 h) of the bacterial growth in BUP medium (Unni et al. 2014). Wensing et al. (2010) reported that maximum siderophore production from *P. syrinagae* was achieved at early stationary phase.

Maximizing the yield of a bioprocess is a crucial factor towards an authenticated and profitable bioprocess. In this regard, the effect of nine physicochemical factors on the production of SID EGY II by *P. aeruginosa* strain EGY II was thoroughly studied through a statistical design; a fractional factorial Plackett–Burman design widely employed in optimization purposes in industrialized bio-processing avoiding the shortcomings encountered in the traditional methods (one variable at a time) (Embaby et al. 2014a, b, 2015). Regarding the effect of pH, no significant effect (P > 0.05) on the production of SID EGY II could be noted. Conversely, pH was reported to be a crucial factor for pyoverdine production by *P. fluorescens*, *P. aeruginosa*, *Alcaligenes faecalis* and *B. amyloliqfaceins* (Albesa et al. 1985; Sayyed et al. 2010; Tailor and Joshi 2012; Bholay et al. 2012; Gaonkar and Bhosle 2013). Pertaining to the influence imposed by incubation temperature on siderophore production by *P. aeruginosa* strain EGYII DSM 101801, no significant impact was noticed. Likewise our finding, it was reported that siderophore production from *P. fluorescens* was achieved between 28 and 30 °C (Tailor and Joshi 2012). However, siderophore production was maximized at 35–37 °C for *A. faecalis* (Sayyed et al. 2010). Form another side, optimal incubation temperature favored for bacterial growth is far away than that required for maximum siderophore production (Bolton et al. 1993). In this context, optimal growth temperatures for *P. aeruginosa* strain EGYII DSM 101801 and *A. faecalis* were 37 and 25 °C, respectively. Regarding carbon source, glucose did not display any significant effect on siderophoregensis by *P. aeruginosa* strain EGYII DSM 101801 likewise the finding of Sayyed et al. (2010). On the other hand, optimal siderophore production from *B. amyloliqfaceins* was reached with glucose addition in the growth medium (Gaonkar and Bhosle 2013). Addition of starch in the production medium imposed a significant effect on siderophoregenesis by *A. faecalis* whilst, siderophore production by *P. aeruginosa* strain EGYII DSM 101801 did influence negatively by starch addition in the production medium. Despite the incapability of *P. aeruginosa* strain EGYII DSM 101801 to utilize starch, the negative influence imposed by starch on SID EGYII production might be attributed to repression of some genes involved in the pathway of siderophoregensis. Regarding agitating speed, an enhanced siderophore production was noticed by increasing agitation speed to 180 rpm. This could be attributed to achieving accelerated growth rates by increasing agitation speed that would in turn result in shorter log phase and an earlier stationary phase. Present finding gives an insight towards scaling up siderophore production from *P. aeruginosa* strain EGYII DSM 101801, where, testing different agitation speeds in the fermentor alongside monitoring the bacterial growth and the siderophore production would be a mandatory task to help mediate a short log phase and achieving to an earlier stationary phase. Time factor is an important factor in the agenda of industrialized bio-processing as long as the shorter the time is required to accomplish a bioprocess, the more reduced cost is encountered. Maximal siderophore production from *P. aeruginosa* strain EGYII DSM 101801 occurred after 24 h of growth. However, siderophore production from other bacteria was achieved from 24 to 96 h, thus, *P. aeruginosa* strain EGYII DSM 101801 is a superior strain when compared to others reported in the literature (Ines et al. 2012; Unni et al. 2014).

At most, employing *P. aeruginosa* strains in industrialized bio-processing is a fuzzy issue raising several questions regarding the likelihood of health risks imposed by such strains even though they are of environmental origin not clinical. This in turn addressed the cardinal need to seek for the location of genes encoding the present unusual siderophore trait in the genome of *P. aeruginosa* strain EGYII DSM 101801. Present finding reveals that the whole genes responsible for siderophoregenesis of this non-fluorescent unusual siderophore by this strain is carried out on a plasmid mediated iron uptake system namely pSID/EGYII as deduced from the perceivable zones of inhibition of *E. amylovora* strain EGY1 DSM 101800 growth mediated by cell lysate of *E. coli* JM109 (DE3) transformants harboring pSID/EGYII. So far, no available reports have handled this issue extensively. Hence, the need to carry out a mini library to catch up the whole genes harbored by the naturally existing pSID/EGYII is highly addressed to gain more insights about the nature of these genes prior to employing the recombinant strain *E. coli* JM109 (DE3)pSID/EGY II in commercialized bio-processing for an intended absolute exploitation of this unusual siderophore in the future. Present finding is in agreement with those of Tolmasky et al. and Osorio et al. who stated that the genes encoding for the anguibactin and piscibactin iron uptake systems in *V. anguillarum* and *Photobacterium damselae* subsp. *Piscicida* are harbored by pJM1and pPHDP70 plasmids, respectively (Tolmasky et al. 1988; Osorio et al. 2015).

Conclusively, SID EGYII is unusual promising non-fluorescent siderophore produced by *P. aeruginosa* strain EGYII DSM 101801; showing broad spectrum antimicrobial activity against Gram-negative bacteria, Gram–positive bacteria and yeast as well. The newly tailored siderophore assay is simple, cheap and good alternate to the currently applied Chromazurol-Shuttle assay. Genes encoding SID EGYII activity were proved to be carried out on a plasmid.

Prospective studies would likely focus on intensive molecular study on pSID EGYII plasmid regarding physical map, size, ori and genes encoding for siderophore activity. Moreover, the chemical structure of this newly siderophore would be detailed studied in the future. Alongside maximizing the productivity of SID EGYII on a large scale using the recombinant *E. coli* JM109 (DE3) pSID/EGYII strain should be studied as well.
